# 550. Bromelain Enzymatic Debridement in Practice: Insights from Real-World Data

**DOI:** 10.1093/jbcr/irag033.247

**Published:** 2026-04-06

**Authors:** Courtney Tresslar, Rohit Mittal, Deanna M DeHoff, Adria Johnson, Ashley B Hink, Steven A Kahn

**Affiliations:** Medical University of South Carolina, Charleston, SC; South Carolina Burn Center at Medical University of South Carolina, Charleston, SC; South Carolina Burn Center at Medical University of South Carolina, Charleston, SC; Medical University of South Carolina, Charleston, SC; Medical University of South Carolina, Charleston, SC; Medical University of South Carolina, Charleston, SC

## Abstract

**Introduction:**

Bromelain-based enzymatic debridement of burn injuries has emerged as a promising alternative to tangential excision. It has been shown to reduce time to wound closure and autografting, and potentially improve scar quality. In December 2022, bromelain-based enzymatic debridement received FDA approval for burns up to 15% total body surface area (TBSA). Use of this debridement technique is still relatively new in the US, and as such, description of its use clinically outside of research study setting is limited. This study aims to describe a single center’s experience utilizing Bromelain-based enzymatic debridement; including patient and injury characteristics, subsequent procedures, and length of stay (LOS).

**Methods:**

This was a retrospective, single institution chart review of adult patients with burns, who underwent bromelain-based enzymatic debridement between June 2020 and March 2025. We assessed patient demographics, injury characteristics, pain management strategies, surgical procedures for wound closure, time interval between bromelain and surgery for wound closure, and number of surgical/laser procedures performed. Our primary endpoint was assessing mean LOS per %TBSA compared to the Burn Care Quality Platform (BCQP) registry.

**Results:**

A total of 53 patients underwent Bromelain-enzymatic debridement with a mean age of 42. Mean TBSA was 17% and mean percent full thickness was 6%. A majority (63.5%) received ketamine orally in the burn step-down unit, while 21.1% received it intravenously. A nerve block was performed in 3.5%, while the remaining 10.5% received an alternative analgesic and sedative strategy. A majority of patients (66%) received skin suspension autograft, while 62% of patients received STSG, 53% polylactic acid sheet skin substitute, and 30% allograft. A small percentage of patients received xenograft skin substitutes. For 28% of patients, a surgical procedure was performed the same day as bromelain debridement; 53% had a procedure the following day. Patients underwent an average of 2 surgical procedures, and 1.1 laser procedures. Lasers were performed as soon as 4 weeks after burn wound closure without complication. Only 1 patient had STSG loss. LOS per %TBSA was 2.1 days with bromelain-based enzymatic debridement, less than the 3.3 days expected based on national BCQP data.

**Conclusions:**

Patients treated with bromelain-based enzymatic debridement had a shorter LOS/%TBSA than expected based on BCQP data (2.1 vs 3.3 days). This study also demonstrates the feasibility of bromelain-enzymatic debridement in the US in clinical settings. Advantages include that oral ketamine can be used for procedural anesthesia for bromelain application and patients can receive surgical wound closure on the same day as bromelain application.

**Applicability of Research to Practice:**

Continued study and refinement of best practices will be necessary to optimize real-world use of bromelain for burn patients and identify optimal recipients.

**Funding for the study:**

N/A.

